# ResponseNet v.3: revealing signaling and regulatory pathways connecting your proteins and genes across human tissues

**DOI:** 10.1093/nar/gkz421

**Published:** 2019-05-22

**Authors:** Omer Basha, Omry Mauer, Eyal Simonovsky, Rotem Shpringer, Esti Yeger-Lotem

**Affiliations:** 1 Department of Clinical Biochemistry & Pharmacology, Faculty of Health Sciences; 2 National Institute for Biotechnology in the Negev, Ben-Gurion University of the Negev, Beer-Sheva 84105, Israel

## Abstract

ResponseNet v.3 is an enhanced version of ResponseNet, a web server that is designed to highlight signaling and regulatory pathways connecting user-defined proteins and genes by using the ResponseNet network optimization approach (http://netbio.bgu.ac.il/respnet). Users run ResponseNet by defining source and target sets of proteins, genes and/or microRNAs, and by specifying a molecular interaction network (interactome). The output of ResponseNet is a sparse, high-probability interactome subnetwork that connects the two sets, thereby revealing additional molecules and interactions that are involved in the studied condition. In recent years, massive efforts were invested in profiling the transcriptomes of human tissues, enabling the inference of human tissue interactomes. ResponseNet v.3 expands ResponseNet2.0 by harnessing ∼11,600 RNA-sequenced human tissue profiles made available by the Genotype-Tissue Expression consortium, to support context-specific analysis of 44 human tissues. Thus, ResponseNet v.3 allows users to illuminate the signaling and regulatory pathways potentially active in the context of a specific tissue, and to compare them with active pathways in other tissues. In the era of precision medicine, such analyses open the door for tissue- and patient-specific analyses of pathways and diseases.

## INTRODUCTION

Signaling and regulatory pathways underlie cellular responses to environmental changes, hormones, stress and other perturbations. Alteration in these pathways could give rise to various diseases, including cancer. Due to their importance, massive efforts were invested in detecting the molecular interactions composing these pathways and other processes by a variety of experimental approaches (1,2). The integration of the detected interactions into a global molecular interaction network, known as an interactome, was shown to illuminate protein functions and cellular processes (3), thereby laying the foundation for the field of network biology (4).

The inference of molecular pathways is challenged by the huge sizes of interactomes. For instance, the interactomes of the heavily studied yeast and human, though still incompletely mapped, are already in the order of 110,000 and 400,000 protein–protein interactions (PPIs), respectively (5). Further complicating pathway inference is the fact that most known interactions were detected in standard or non-physiological conditions, making them context-less, while the pathways themselves are highly context specific. Across the years, these challenges were met by numerous approaches aimed at identifying linear and complex pathways that are meaningful in the studied context by, e.g. learning from known pathways and applying context-based filtering (6,7).

In recent years, analysis of tissue contexts has been greatly facilitated owing to large-scale tissue profiling efforts, such as the Genotype-Tissue expression (GTEx) (8) and the Human protein Atlas (9). To date, GTEx is one of the largest resources, containing ∼11,600 RNA-sequenced tissue profiles from over 40 human tissues. The integration of tissue profiling data with the context-less human interactome allowed for construction of tissue interactomes, where interactions were weighted according to the expression of the interacting partners in the respective tissue (10). The resulting tissue interactomes were shown to outperform the global interactome in prioritizing disease genes (11), and to illuminate protein functions (12) and disease mechanisms (13,14).

ResponseNet is an integrative approach designed to identify a high-probability interactome subnetwork connecting user-defined proteins to their downstream affected genes (15). Formulated as a minimum cost-flow optimization problem that is solved by linear programming, ResponseNet outputs a sparse, high-probability subnetwork that the user can grasp and subsequently define testable hypotheses. ResponseNet was originally applied and tested on a yeast model of synucleinopathy related to Parkinson's disease, where it revealed pathways that were validated experimentally (15). Later implemented as a web server (16) and extended to support analysis of human data (17), ResponseNet was successfully used to infer pathways in various contexts, e.g. to establish the subnetwork acting downstream of the Rio1 kinase in yeast (18), to predict pathways involved in intellectual and behavioural disabilities (19) and to identify signaling pathways responding to bacterial infection (20).

Several other approaches were introduced to infer causal pathways, in the form of interactome subnetworks connecting sets of proteins and genes associated with a certain phenotype or disease. Such methods were formulated as, e.g. Prize-collecting Steiner trees (21), network flow (22), integer programming (23) or electrical circuits (24). Several approaches were provided as open software, such as iPoint (25) and Omics Integrator (26), or as webservers (27,28), making them accessible to researchers.

In recent years it became clear that multiple traits and phenotypes manifest in a tissue-selective manner. Mendelian diseases tend to be highly tissue-specific (13,19,20,29,30), and variation in genomic sequences were shown to underlie tissue-specific altered gene expression and complex traits (31). To help uncover the pathways underlying tissue-selective traits and diseases, we expanded the ResponseNet web server toward analysis of pathways in tissue contexts. By integrating data from GTEx (8), we created tissue interactomes for 44 tissues, enabling their analysis via ResponseNet. To facilitate the interpretation of the output network and the design of follow-up experiments, we augmented the output with data of disease-related proteins and protein targets of approved drugs. Lastly, we added graphical network comparisons, making it feasible to compare between subnetworks predicted for different tissues or between individual runs with slightly distinct inputs, thereby facilitating the interpretation of genotype–phenotype relationships.

## RESULTS

**Figure 1.**
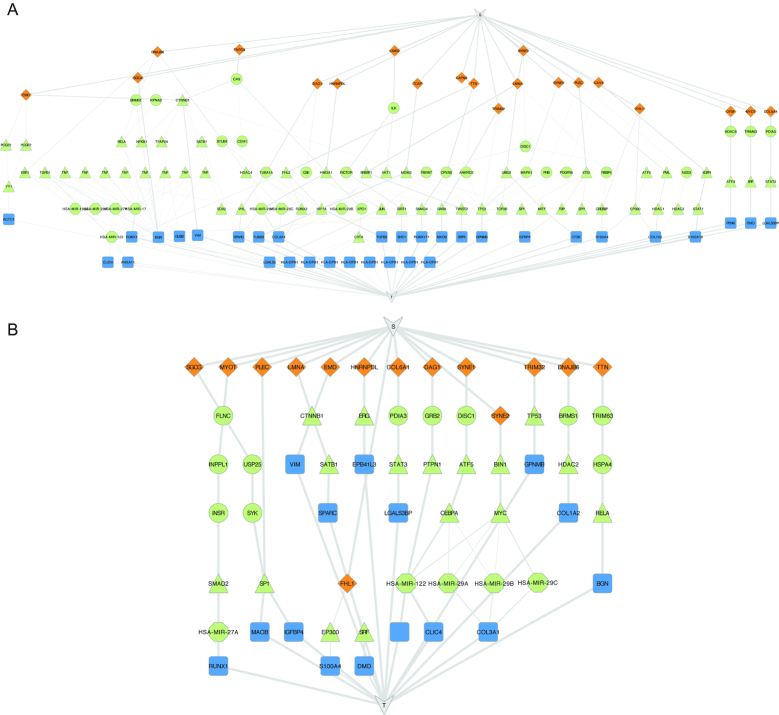
ResponseNet v.3 output and subnetwork comparisons. (**A** and **B**) The subnetworks predicted by ResponseNet as connecting between proteins that are causal for muscular dystrophy (diamond-shaped node) and genes that were differentially expressed in muscle biopsies of patients versus healthy controls (rectangle-shaped nodes). (A) Subnetwork predicted by using the global interactome, containing 86 connecting proteins and microRNAs. (B) Subnetwork predicted by using the skeletal muscle interactome, containing 33 connecting proteins. This subnetwork contained several unique pathways, including the path connecting DNAJB6 to COL1A2, and the predicted involvement of miR-29 in the regulation of COL3A1, described in the text.

To identify signaling and regulatory pathways by using the ResponseNet webserver, the user defines two sets: a source set containing proteins, and a target set containing genes, proteins or microRNAs. The two sets can vary in size from few to hundreds. Examples include analysis of disease-causing mutations and genes that are differentially expressed in patients (18); the proteins affected by a virus and the host response to viral infection (32,33); or the results of a genetic screen and the cellular response to perturbation (15). The user then selects the interactome to be searched, or uploads a weighted interactome. The output includes a weighted interactome subnetwork, connecting a subset of the source set to a subset of the target set via high-probability pathways. In case the target set is composed of genes and/or microRNAs, the connecting pathways are composed of PPIs and end with a regulatory interaction, thereby illuminating signaling and regulatory pathways (Figure 1A and B). More details about the performance of ResponseNet and its usability can be found elsewhere (17). Below we focus on the features we introduced in ResponseNet v.3.

### Construction of human tissue interactomes

ResponseNet v.3 integrated data of molecular interactions and tissue transcriptomic profiles to create human tissue interactomes. For this, we first gathered experimentally detected molecular interactions involving human proteins, genes and microRNAs. Data of PPIs were gathered from BioGRID (5), MINT (34) and IntAct (35) by using the MyProteinNet web server (36). MyProteinNet was used to ensure that only PPIs detected by established experimental methods were included, and to associate interactions with probabilistic weights, such that interactions participating in known response pathways were favored (15). Data of regulatory interactions between transcription factors (TFs) and their target genes were downloaded from TRRUST (37) and TransmiR (38). Regulatory interactions between microRNAs and their target transcripts were downloaded from miRecords (39). These interactions were combined into a global human interactome containing 327,408 interactions between 22,264 molecules (Table 1).

**Table 1.** Numbers of molecular interactions in the global human interactome

Interaction type	Proteins	Genes	microRNAs	Interactions
PPI	18,178			311,737
TF–DNA	855	2,937		10,500
TF–microRNAs	372		402	2,369
microRNAs–RNA		996	267	1,582
Total	18,189	3,540	535	327,408

To create tissue interactomes, we integrated the global human interactome with over 11,200 RNA-sequenced profiles of 44 tissues, made available by the GTEx consortium (8). The interactome of a specific tissue was created via a filtering scheme (13), such that only PPIs in which both pair mates were expressed in that tissue above a certain threshold were included (See ‘Methods’, (40)). Interactions involving microRNAs were not filtered. This resulted in interactomes for 44 tissues containing on average 223,052 interactions between 13,776 proteins, 535 microRNAs and 3,540 genes. Tissue interactomes can be downloaded from the ResponseNet download page. Note that since the interactomes do not contain all human genes, some of the input proteins and genes might not be connected via ResponseNet.

### User session maintenance and email notifications

ResponseNet algorithm is executed on dedicated servers. Due to interactome sizes, run times can take up to 35 min for a single global interactome run and about 5 min for tissue-specific interactomes, and may take longer if randomizations are carried (17). Users are therefore encouraged to specify a user name in ResponseNet to save their sessions. Sessions will be kept for 3 months. ResponseNet v.3 allows users to provide an email address, through which they will be notified once their run is complete.

### New usability features

The output of ResponseNet includes, per gene, its description and GO annotations and per interaction, its detection methods. To this we added several new features aimed to facilitate the interpretation of the output network and experimental planning. To enhance interpretation and to identify potentially relevant diseases, genes associated with Mendelian diseases were distinctly marked as such in the graphical network, and disease information was provided in the properties tab. To facilitate the planning of follow-up experiments, protein targets of approved drugs were gathered from DrugBank (41) and were listed in the properties tab for applicable genes. Since drug effectiveness could be lower for genes whose expression varies across individuals (42), a score representing this variability (with zero for non-variable and 100 for maximum variability) also appears, thereby facilitating candidate prioritization.

### Subnetwork comparison

ResponseNet v.3 also allows users to compare between subnetworks. Such subnetworks could represent the pathways inferred in distinct tissues, or in individuals with distinct genotypes or phenotypes. ResponseNet offers a graphical layers mechanism to support subnetwork comparisons. The user can define a layer by selecting a subset of the output network, by selecting subnetworks from previous sessions, or by importing a network from other runs by using the export option. Once a layer was defined, the user can name it and compare it to other layers using union, intersection, difference or XOR operations. By that, pathways that are common to multiple layers (e.g. multiple tissues or individuals), or pathways that are unique to a certain layer can be immediately recognized.

### Use case

To demonstrate the power of the ResponseNet v.3 server and the tissue-specific networks we analyzed the signaling and regulatory networks that might be involved or disrupted in muscular dystrophy. To define the source set, we gathered from the OMIM database 34 genes known to cause muscular dystrophy. To define the target set, we used 97 genes that were found to be differentially expressed in biopsies of Duchenne muscular dystrophy patients relative to healthy controls (43). We then applied ResponseNet to find pathways that connect the two sets in the global human interactome (Figure 1A) and in the interactome of skeletal muscle (Figure 1B). The output subnetwork predicted by ResponseNet for skeletal muscle was smaller than the subnetwork predicted for global interactome (33 versus 86 connecting proteins and microRNAs) and offered several potential pathways. For example, it identified a pathway connecting the source protein DNAJB6, a chaperone that is causal gene for limb-griddle muscular dystrophy (44), and the target gene COL1A2 that was shown to be upregulated in muscular dystrophy patients (43). ResponseNet positioned DNAJB6 upstream of BRMS1, which it is known to stabilize (45), and BRMS1 upstream of HDAC2. BRMS1 and HDAC2 are part of a histone deacetylase complex (HDAC), and the predicted disruption of this pathway indeed fits with studies that linked muscular dystrophies to deregulated HDAC activity (46). Another pathway placed the COL3A1 gene, which was shown to be upregulated in muscular dystrophy (43), downstream of miR-29, whose loss was indeed connected to dystrophic muscle pathogenesis (47). ResponseNet also suggested that a problem in the SYNE2 gene, which is known to cause Emery Dreifuss muscular dystrophy, leads to the loss of miR-29 by affecting the MYC transcription regulation complex. Notably, these pathways were not predicted in the global interactome, stressing the advantage of using context-specific networks.

## SUMMARY

The ResponseNet v.3 web server enables users to apply the ResponseNet network optimization approach to infer signaling and regulatory pathways active in tissue contexts. In addition to features supported by previous versions of ResponseNet, ResponseNet v.3 highlights disease-related proteins and drug targets, and provides a graphical network comparison tool. ResponseNet v.3 functionality is easily expandable to additional tissues, cell types and other contexts. Tools such as ResponseNet that can provide meaningful views into the pathways underlying traits and diseases (31) and their therapeutic manipulation (48), are highly timely in light of the increasing availability and declining cost of genomic and transcriptomic profiling, and their enhanced usage in clinical settings.

## METHODS

### Expression data sources

RNA sequencing profiles were obtained from the GTEx portal (version 7) (8), resulting in 11,216 samples from 44 tissues. Only genes with more than five read counts in at least 10 samples were included in the analysis. Raw read counts were normalized for sample library size via the TMM method by edgeR (49) to produce counts per million (cpm). Only genes with cpm values ≥ 8 in at least 10 samples were considered henceforth. Samples per tissue were merged such that the expression of each gene was set to its median expression value across samples. Only genes with a median cpm log2 value ≥ 8 were considered as expressed in the respective tissues.

### Protein–protein interactions data and weighting scheme

Human and Yeast PPIs were gathered from BioGrid (5), MINT (34) and IntAct (35) by using the MyProteinNet web-server (36). MyProteinNet ensures that only PPIs detected by established methods for physical interactions detection were considered. Moreover, it associates interactions with weights that represent their reliability and tendency to appear in response pathways. Human transcription regulation interactions between TFs and their target genes were downloaded from the TRRUST (37) on 16 April 2018. Transcription regulation interactions between TFs and their target microRNAs were downloaded from the TransmiR database v.2 (38). Experimentally detected interactions between microRNAs and their target transcripts were downloaded from the miRecords database (39). All transcription regulation interactions (TF–DNA, TF–microRNA and microRNA–RNA) were assigned a weight of 1. To consolidate the interactions from the different databases, all identifiers were converted to Ensemble gene IDs using the MyGene.info web-service (50) and combined into one interactome. Each node in the interactome was defined by its identifier and its type (protein, gene or microRNA), thereby allowing for distinct nodes for a gene and its product.

### Additional supporting data

Associations between diseases and their causal genes were downloaded from the OMIM database on January 2018 (51). Data of approved drugs and their protein targets were downloaded from the DrugBank database on December 2018 (41). Expression variability scores per gene were obtained from Simonovsky *et al.* (42). Data of genes that were differentially expressed in patients with Duchenne muscular dystrophy versus controls were obtained from Haslett *et al.* (43).

### Implementation

The ResponseNet v.3 server was implemented in Python by using the Flask framework with data stored on a MySQL database. The website client was programmed using the ReactJS framework and designed with Semantic-UI. The network view is displayed by the cytoscape.js plugin (52). The website supports all major browsers. Recommended viewing resolution is 1440 × 900 and above.

### Download

Interactomes are available for download under the permissive Creative Commons Attribution License (http://creativecommons.org/licenses/by-nc/4.0/), which permits non-commercial re-use, distribution and reproduction in any medium, provided this work is properly cited. For commercial use, please contact the corresponding author. Downloadable data are versioned by numbered tissue interactomes builds and by global interactome build dates. The download page enables users to download data separately for each tissue.

### Web service access

The ResponseNet v.3 web-server supports a web-service access to programmatically download previous sessions. This is implemented via a REST-API method, which is callable via code or wget. More information can be found at http://netbio.bgu.ac.il/respnet-api.
